# Short-term outcomes in early term infants (born at 37 or 38 weeks): a retrospective investigation

**DOI:** 10.3389/fped.2024.1430364

**Published:** 2024-10-10

**Authors:** Tsubasa Kitamura, Kyosuke Tabata, Yayoi Murano, Daisuke Yoneoka, Tomoyuki Nakazawa, Ken Sakamaki, Hiromichi Shoji

**Affiliations:** ^1^ Division of Obstetrics and Gynecology, Tokyo Metropolitan Toshima Hospital, Tokyo, Japan; ^2^ Division of Pediatrics, Tokyo Metropolitan Toshima Hospital, Tokyo, Japan; ^3^ Department of Pediatrics, Juntendo University Faculty of Medicine, Tokyo, Japan; ^4^ Center for Surveillance, Immunization, and Epidemiologic Research, National Center of Infectious Disease, Tokyo, Japan

**Keywords:** early term, infants, logistic regression, cesarean section, term delivery, outcomes

## Abstract

**Introduction:**

Recently, researchers have introduced the concept of ‘early term’ infants, defined as infants born at 37 or 38 weeks of gestation, and their outcome has been discussed. Although the complications experienced by early term are less severe than those in preterm infants, this group accounts for a much larger proportion of newborns, making the assessment of outcomes important in clinical practice.

**Methods:**

This observational study of term infants born at Tokyo Metropolitan Toshima Hospital aimed to understand the short-term outcomes in early term infants. Data extracted from the medical records were analyzed.

**Results:**

Among 4,669 eligible participants, 463 (9.9%) were born at 37 weeks and 1,270 (27.2%) were born at 38 weeks. The remaining 2,936 infants were born after 39 weeks of gestation. Logistic regression analysis showed higher odds ratio of hospitalization (1.56, 95% CI: 1.37–1.79, *p* < 0.05), apnea (2.23, 95%CI: 1.08–4.60, *p* < 0.05), and hypoglycemia (3.13, 95%CI: 1.95–5.03, *p* < 0.05) in early term infants. In detail, infants born at 37 weeks of gestational age had higher odds ratio for hospitalization (2.07, 95%CI: 1.68–2.35, *p* < 0.05) and hypoglycemia (4.11, 95%CI 2.22–7.60, *p* < 0.05) than infants born at 38 weeks of gestational age (1.40, 95%CI: 1.20–1.62, *p* < 0.05, and 2.78, 95%CI: 1.66–4.67, *p* < 0.0 respectively).

**Discussion:**

This study revealed complicated clinical course in early term infants, and represents one of the largest contributions to understanding the outcomes of early term infants, and could help to determine strategies for elective cesarean section. According to this result, elective cesarean sections would be better planned at 38 weeks of gestational age. Moreover, in clinical practice, it is important to be aware of the complicated clinical course in early term infants.

## 1 Introduction

Perinatal complications in preterm infants and the importance of follow-up have been well established (1–4). During the past few decades, the outcomes of late preterm infants, who were believed to be near-term and to have fewer perinatal complications, have been reconsidered. Nowadays, this population is known to have a poor outcome compared to term infants, as they do not experience the critical period of their last six weeks in the uterus (5–8). Recently, another concept, classified as ‘early term’ infants has been proposed. This population comprises infants born between 37 and 38 weeks of gestation. Although this population has been found to be at a higher risk of developing perinatal complications compared to infants born after 39 weeks of gestational age (9), relatively few studies have assessed their outcomes. However, an increase in this population has been reported (10), and as early term infants account for a much larger population than preterm infants (9), understanding perinatal outcomes in early term infants is important in clinical practice. Infants born at early term are typically managed in the obstetrics ward, neonatologists do not provide them with routine care, and they are not monitored in the same manner as preterm infants. Consequently, their detailed clinical features are not well-known.

This study therefore investigated the short-term outcomes of early term infants.

## 2 Methods

### 2.1 Study participants

This observational study was conducted retrospectively using data extracted from medical records. The study participants were term infants (born between 37 weeks 0 days of gestation and 41 weeks 6 days of gestation) born between 2015 and 2022 at the Tokyo Metropolitan Toshima Hospital, a secondary hospital in the Tokyo Metropolitan area that manages deliveries performed after 35 weeks of gestational age. All birth data between the study periods were extracted, and individual data on gestational age, sex, birth weight, maternal age, Apgar score, cesarean section (yes/no), and hospitalization after birth (yes/no, and if yes, reason for hospitalization) were extracted anonymously. ‘Hospitalization’ was defined as patients admitted to the growth care unit of the pediatric ward. The pediatrician performed examinations and made decisions whenever the attending gynecologist requested a consultation.

Gestational weeks were calculated based on the recommendations of the Japan Association of Obstetricians and Gynecologist in Japan. Firstly, if there is precise information regarding fertilization, including data on basal body temperature, limited intercourse, artificial insemination, or *in vitro* fertilization, the test day was classified as 2 weeks 0 days of gestation. Secondary, if the menstrual cycle was regular and lasted from 28 to 30 days and if the date of the last menstrual period was known accurately, the first day of the last menstrual period was considered 0 week 0 day of gestation. If neither of the following information was available, a crown-rump length of 15–30 mm or a biparietal diameter of 20–30 mm was used to determine gestational age.

The reasons for hospitalization and their criteria were as follows: neonatal jaundice (jaundice that requires phototherapy), feeding disorder (mal-feeding requiring hospitalization), transient tachypnea of the newborn (respiratory disorder without pneumonia, pneumothorax), meconium aspiration syndrome (respiratory disorder with meconium aspiration), hypoglycemia (blood glucose less than 40 mg/dl that does not improve with feeding), acute gastric mucosal lesions (bloody vomiting), melena bloody stools), asphyxia (diagnosed by attending pediatrician and requiring hospitalization), vomiting (vomiting requiring management), neonatal fever (significant fever over 38°C), and, apnea (respiratory arrest for more than 30 s or complicates bradycardia). To diagnose respiratory disorders, we used chest x-ray results, which were examined by a blinded pediatrician, and transient tachypnea and meconium aspiration syndrome were clearly discriminated. Pneumonia was diagnosed based on consolidation on radiographs.

First, the participants were limited to term infants based on gestational age data. Early term was defined as birth between 37 weeks, 0 days, and 38 weeks, and 6 days of gestational age.

### 2.2 Statistical methods

Comparisons between early term (born before 38 weeks) and other term infants (born after 38 weeks and 0 days of gestation) were performed. The same procedure was then performed separately for the groups of infants born in the 37th and 38th weeks of gestation.

When analyzing the participants’ characteristics, the *t*-test was used for continuous variables and the chi-square test was used for categorical variables. Logistic regression was used to analyze the association between hospitalization (yes or no) and each reason for hospitalization, and early term or 37 and 38 weeks of gestational age were analyzed separately. Statistical analyses were performed using Stata software version 15.1 (Stata Corp, 4905 Lakeway Drive College Station, TX 77845, USA). Statistical significance was set at *p* < 0.05.

### 2.3 Ethical approval

This study was approved by the Ethics Committee of Tokyo Metropolitan Toshima Hospital (approval number: Jin 4–17). The requirement for patient consent was waived owing to the use of anonymous data.

## 3 Results

Among the 4,821 infants born during the study period, 151 were preterm (born before 37 weeks of gestational age) and one was born after 42 weeks of gestational age. After excluding these infants, the remaining 4,669 infants were included in the present study. Among these, 463 (9.9%) were born at 37 weeks, 1,270 (27.2%) at 38 weeks, 1,400 (29.9%) at 39 weeks, 1,189 (25.4%) at 40 weeks, and 347 (7.4%) at 41 weeks of gestation. The total number of early term infants, defined as those born between 37 and 38 weeks of gestational age, was 1,733 (37.1%).

Table 1 shows the characteristics of the participants, comparing the early term and term infants. Significant differences were observed in sex, birth weight, maternal age, proportion of cesarean sections, and infant hospitalization. Table 2 shows the comparison of the characteristics of the participants born at 37 and 38 weeks of gestational age. There were no significant differences in maternal age, Apgar scores, or proportion of cesarean section births between the two groups. However, birth weight, proportion of elective cesarean section in cesarean section, and infant hospitalization rate differed significantly.

**Table 1:** Basic characteristics of early term and term participants (Early term vs. term).

	Early term (37–38 weeks of gestational age) (*n* = 1,733)	Term infants (39–41 weeks of gestational age) (*n* = 2,936)	*p*-value
Sex [boy (%)]^a^	910 (52.5)	1,439 (49.0)	0.02
Birth weight (g) (mean ± SD)^b^	2,912 ± 349	3,150 ± 352	0.00
Maternal age (years old) (mean ± SD)^b^	32.2 ± 5.2	31.0 ± 5.3	0.00
Apgar score 1 min (mean ± SD)^b^	8.1 ± 2.8	8.2 ± 0.9	0.27
Apgar score 5 min (mean ± SD)^b^	8.9 ± 2.7	8.9 ± 0.6	0.41
Cesarean section (%)^a^	559 (32.2%)	322 (11.0%)	0.00
Proportion of elective cesarean section (%)^a^	452/559 (80.9%)	11/322 (3.4%)	0.00
Infant hospitalization^b^	518 (29.9%)	629 (21.4%)	0.00

SD, standard deviation.

^a^ Calculated using the chi-square test.

^b^ Calculated using the *t*-test.

**Table 2:** Comparison of characteristics between infants born at 37 and 38 weeks of gestational age.

	37 weeks gestational age	38 weeks gestational age	*p*-value
	(*n* = 463)	(*n* = 1,270)	
Sex [boy (%)]^a^	239 (51.6)	671 (52.8)	0.65
Birth weight (g) (mean ± SD)^b^	2,801 ± 314	2,952 ± 352	0.00
Maternal age (years old) (mean ± SD)^b^	32.5 ± 5.2	32.1 ± 5.1	0.16
Apgar score 1 min (mean ± SD)^b^	8.2 ± 1.2	8.1 ± 3.2	0.81
Apgar score 5 min (mean ± SD)^b^	8.9 ± 0.9	8.9 ± 3.1	0.92
Cesarean section (%)^a^	164 (35.4%)	395 (31.1%)	0.09
Proportion of elective cesarean section (%)^a^	120/164 (73.1%)	332/395 (84.1%)	0.0003
Infant hospitalization^b^	142 (30.1%)	285 (22.4%)	0.000

SD, standard deviation.

^a^ Calculated using the chi-square test.

^b^ Calculated using the *t*-test.

The odds ratios for hospitalization were analyzed using univariate logistic regression. As shown in Table 3, the odds ratios for hospitalization, apnea, and hypoglycemia were significantly higher in early term infants.

**Table 3:** Results of logistic regression.

	Odds ratio	95% C.I	*p*-value
Hospitalization	**1**.**38**	**1.19**–**1.59**	**0**.**00**
Neonatal jaundice	1.11	0.90–1.37	0.32
Feeding disorder	1.27	0.28–5.69	0.75
Transient tachypnea of the newborn	1.67	1.18–2.36	0.00
Meconium aspiration syndrome	0.21	0.06–0.70	0.01
Hypoglycemia	**4**.**54**	**3.06**–**6.74**	**0**.**00**
Acute gastric mucosal lesions	0.48	0.16–1.47	0.20
Melena	0.48	0.16–1.47	0.20
Asphyxia	0.59	0.35–1.00	0.051
Vomiting	0.95	0.42–2.16	0.91
Neonatal fever	0.34	0.10–1.17	0.08
Apnea	**3**.**13**	**1.55**–**6.34**	**0**.**00**

Determining odds ratio for each outcome in early term infants are shown.

Table 4 shows the results of the analysis of outcomes of early term infants born at 37 and 38 weeks of gestational age, and those of infants born after 38 weeks of gestational age. The odds ratios for hospitalization and hospitalization for hypoglycemia and apnea were higher in infants born at 37 and 38 weeks of gestation compared to those born later, while both factors were higher in infants born at 37 weeks of gestation than in infants born after 38 weeks of gestation. Feeding disorder and transient tachypnea of the new born had higher odds ratios only in 37 weeks of gestation, while odds ratio was higher in asphyxia only in 38 weeks of gestation.

**Table 4:** Results of logistic regression.

	37 weeks gestational age			38 weeks gestational age		
	Odds ratio	95% C.I	*p*-value	Odds ratio	95% C.I	*p*-value
Hospitalization	1.86	1.50–2.31	0.00	1.21	1.04–1.43	0.02
Neonatal jaundice	1.10	0.78–1.54	0.53	1.12	0.89–1.40	0.35
Feeding disorder	4.78	1.07–21.43	0.04	N.A		
Transient tachypnea of the newborn	2.31	1.43–3.71	0.00	1.44	0.98–2.13	0.07
Meconium aspiration syndrome	N.A			0.29	0.09–0.96	0.04
Hypoglycemia	7.41	4.63–11.86	0.00	3.54	2.29–5.46	0.00
Acute gastric mucosal lesions	N.A			0.66	0.22–2.01	0.46
Melena	N.A			0.66	0.22–2.01	0.45
Asphyxia	0.82	0.37–1.81	0.62	0.51	0.27–0.95	0.03
Vomiting	1.19	0.35–4.10	0.78	0.87	0.34–2.22	0.77
Neonatal fever	N.A			0.46	0.13–1.60	0.22
Apnea	3.74	1.47–9.55	0.01	2.91	1.36–6.24	0.01

The odds ratios for each outcome in infants born at 37 and 38 weeks of gestation compared to infants born beyond 39 weeks of gestation.

## 4 Discussion

The present study showed complicated clinical course in infants born in the early term compared with those born after 38 weeks of gestation. The former group had higher odds ratios for hospitalization, apnea, and hypoglycemia. Further analysis distinguishing between early term infants born at 37 and 38 weeks gestation revealed more complicated clinical outcomes in infants born at 37 weeks gestation.

In Japan, the proportion of early term births has increased in recent years. The most important reason for this is the establishment for indications for elective cesarean section, with infants in the breech position, mothers with a history of cesarean section, and mothers with a history of uterine surgery all included as indications for cesarean section (11–13). Elective cesarean sections can be planned at gestational ages of 37–38 weeks, or 39–40 weeks of gestational age (14, 15). As hospitals and clinics can be scarce throughout Japan, with only one gynecologist staying on call at night, cesarean sections are commonly planned between 37 and 38 weeks of gestational age. As a result, the proportion of infants born at early term (between 37 and 38 weeks of gestational age) is increasing. Therefore, understanding the outcomes at these gestational ages is important (16).

In regards elective cesarean section, the results of the present study showing better outcome in infants born at 38 weeks of gestational age compared to those born at 37 weeks of gestational age, it could be hypothesized that planning elective cesarean section for 38 weeks of gestational age instead of 37 would contribute to better outcome. However, we did not analyze data about cases of elective cesarean section shifting to emergency cesarean section, and the outcome has not been discussed. As such, further evaluation is required.

Several previous studies have reported poor outcome in infants born at an early term and/or at 37 weeks of gestational age (7, 15); our study results corroborate these reports. However, while some studies have shown an increase in respiratory disorders in early term infants (16), the present study did not. Our study further showed a relatively high odds ratio in early term infants, and when infants born at 37 and 38 weeks of gestation were analyzed separately, those born at 37 weeks of gestation showed higher odds ratios for apnea, though not significantly. This may have been due to the small sample size. However, to the best of our knowledge, an increased risk of apnea in early term infants has not yet been reported. Apnea occurs because of prematurity and several other reasons and is said to be a risk factor for sudden unexpected infant death (SUID) and a severe apparent life-threatening event (ALTE) (17). Unlike preterm infants, early term infants rarely undergo continuous monitoring of their vital signs; however, clinical practitioners should be aware of this complication. Another complication with a higher odds ratio in early term infants is hypoglycemia. As all the participants of this study were term infants, this event was most likely caused by transitional neonatal hypoglycemia (18); however, as prematurity is a known risk factor for hypoglycemia, this remains a possible cause of hypoglycemia. The higher odds ratio in infants born at 37 weeks compared with those born at 38 weeks of gestation supports this idea. Moreover, hospitalization is associated with lower breastfeeding rate (19) which is a major risk factor for SUID (20, 21). Health care practitioners should be aware of this long-term outcome, too.

The most important limitation of this study is that it was a single-center study with a small sample size; however, this had benefits over using a database, as we were able to examine the details of the medical records and extract each diagnosis clearly. Further, in this study, we suggested a better outcome in the electric cesarean section at 38 weeks of gestation than at 37 weeks of gestation. However, we could not assess all outcomes, including those associated with emergency cesarean sections; as such, further evaluation is required. Furthermore, there are several potential confounding variables that should be considered. First, the date of electric cesarean section was selected by attending gynecologists, with surgeries limited to weekdays. This may have influenced the gestational days and requires further evaluation. Another factor is that the indications for hospitalization may depend on the attending pediatricians. To address this confounder, further research with larger sample sizes is required.

## 5 Conclusion

Overall, the results of this study show that early term infants have complicated neonatal outcomes, including higher risks of hospitalization, apnea, and hypoglycemia, compared to infants born after 39 weeks of gestation. Among early term infants, those born at 37 weeks of gestation have a worse outcome than those born at 38 weeks of gestation.

## Data Availability

The raw data supporting the conclusions of this article will be made available by the authors, without undue reservation.
